# Experimental and theoretical study on the driving force and glass flow by laser-induced metal sphere migration in glass

**DOI:** 10.1038/srep38545

**Published:** 2016-12-09

**Authors:** Hirofumi Hidai, Jun Wada, Tatsuki Iwamoto, Souta Matsusaka, Akira Chiba, Tetsuo Kishi, Noboru Morita

**Affiliations:** 1 Department of Mechanical Engineering, Chiba University, 1-33 Yayoi-cho, Inage-ku, Chiba 263-8522, Japan; 2 Department of Materials Science and Engineering, Tokyo Institute of Technology, 2-12-1 Ookayama, Meguro-ku, Tokyo 152-8550, Japan

## Abstract

Light is able to remotely move matter. Among various driving forces, laser-induced metal sphere migration in glass has been reported. The temperature on the laser-illuminated side of the sphere was higher than that on the non-illuminated side. This temperature gradient caused non-uniformity in the interfacial tension between the glass and the melted metal as the tension decreased with increasing temperature. In the present study, we investigated laser-induced metal sphere migration in different glasses using thermal flow calculations, considering the temperature dependence of the material parameters. In addition, the velocity of the glass flow generated by the metal sphere migration was measured and compared with thermal flow calculations. The migration velocity of the stainless steel sphere increased with increasing laser power density; the maximum velocity was 104 μm/s in borosilicate glass and 47 μm/s in silica glass. The sphere was heated to more than 2000 K. The temperature gradient of the interfacial tension between the stainless steel sphere and the glass was calculated to be −2.29 × 10^−5^ N/m/K for borosilicate glass and −2.06 × 10^−5^ N/m/K for silica glass. Glass flowed in the region 15–30 μm from the surface of the sphere, and the 80-μm sphere migrated in a narrow softened channel.

Light is able to remotely move matter^1^. Various driving forces have been reported to cause this migration. A well-known driving force is radiation pressure. When the direction of the light progression is changed, pressure is applied as a counter force to the light. The pressure can be applied to manipulate various kinds of matter, including transparent matter^2^, opaque materials^3^, and nanoparticles^4^.

Other driving forces have also been reported. The photophoretic force also induces the migration of particles, and its direction is dependent on the particle size^5^. Negative photophoresis occurs when particles of a certain diameter migrate in the direction counter to the light progression^6^. Laser scanning generates a liquid flow in molten ice^7^ or in gel^8^ caused by volume changes induced by repetitive melting and freezing.

In contrast to these migration techniques, we have reported laser-induced metal sphere migration in glass^9^. We have revealed the driving mechanism^10^,^11^, nanoparticle precipitation in the trajectory of migration^12^ and the kinds of metal spheres that could be implanted into glass^9^. The driving force of metal sphere migration can be explained as follows: the metal sphere absorbs laser light and converts the energy into heat. The surrounding glass is then heated by conduction and becomes fluid. The temperature on the laser-illuminated side of the sphere is higher than that on the non-illuminated side. This temperature gradient causes a non-uniformity in the interfacial tension between the glass and the melted metal sphere as the tension decreases with increasing temperature. The balance between the driving force arising from the interfacial tension and the drag force arising from the sphere migration determines the migration velocity. Theoretical calculations have estimated that the driving force generated by the interfacial tension is in the order of a few micronewtons^10^,^11^. In these calculations, the material parameters were applied as constant values, and the temperature dependence of the values was ignored. The migration was dominated by the temperature gradient under high temperatures above the glass softening point. In particular, the glass viscosity changed drastically with increasing temperature, affecting the drag force. Hence, the thermal flow calculation, which considers the temperature dependence of the material parameters, is indispensable to reveal the migration mechanism.

Metal sphere migration involves the flow of glass around a metal sphere. The glass is transparent, and visualisation of the flow is difficult. However, metal particles with diameters of a few hundred nanometres precipitate around the metal sphere in silica glass^12^. Particle image velocimetry (PIV) of the precipitated particle enables visualisation of the velocity of glass flow. The measured velocity and calculated velocity can be compared to evaluate the accuracy of theoretical calculations.

In the present study, we investigated laser-induced metal sphere migration in different glasses using thermal flow calculation, considering the temperature dependence of the material parameters. In addition, the velocity of glass flow generated by the metal sphere migration was measured and compared with the thermal flow calculation.

## Theoretical Calculations

The thermal flow of glass around the stainless steel sphere was simulated with commercial software (Solidworks Flow simulation). In the simulation, the temperature dependence of the material parameters was considered. Based on the calculated temperature and pressure, the driving force and the drag force applied to the stainless steel sphere were calculated.

### Thermal flow analysis

Thermal flow around the stainless steel sphere was calculated based on the heat conduction equation and the incompressible Navier–Stokes equation. Temperature *T* [K] and velocity *V* [m/s] of the glass at time *t* [s] were expressed by:


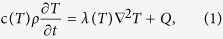



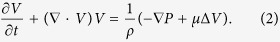


Here, *c*(*T*) [J/kg/K] is the specific heat, *ρ* [kg/m^3^] is the density, *λ*(*T*) [W/m/K] is the thermal conductivity, *μ*(*T*) [Pa·s] is the viscosity, *Q* is the heat source by laser and *P* is the pressure. *c*(*T*)*, λ*(*T*) and *μ*(*T*) are the functions of the temperature.

**Figure 1:**
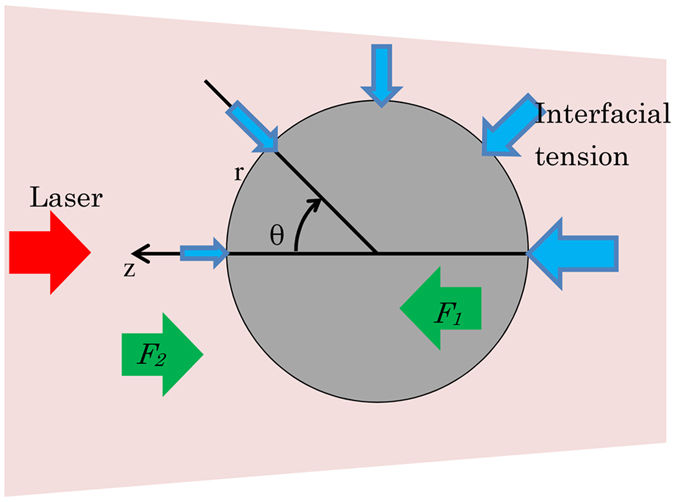
Schematic drawing of the simulation model.

One side of the stainless steel sphere was heated by the laser. The absorption coefficient of stainless steel with a similar composition (SUS316) was reported^13^ to be 5.45 × 10^5^ cm^−1^, so the penetration depth was calculated to be 20 nm, which was much smaller than the diameter of the sphere. Hence the laser was assumed to be absorbed at the sphere surface. The beam diameter, assuming a constant coordinate system, was set as shown in Fig. 1, and the centre of the sphere was set as the origin. The heat source *Q* was expressed as:


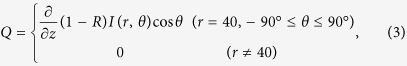



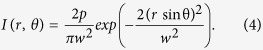


Here, *R* is the reflectivity, *I*(*r*, θ) [W/m^2^] is the beam intensity, *p* [W] is the laser power and *w* [m] is the beam radius. The radius of the sphere was set as 40 μm to match with the experimental results. Here, the laser power density was calculated by the beam intensity divided by the calculated beam radius (1/e^2^).

The time step was set at 1 μs. The temperature increased with an increasing number of steps and eventually became constant. The calculation result after the temperature became steady is discussed later. The steady-state temperature was defined as when the maximum temperature difference was less than 1 K for a subsequent time step. A detailed explanation is provided in the Supplementary information.

### Drag force

By softening the surrounding glass, the heated stainless steel sphere migrates towards the light source at a constant velocity. The drag force applied to the migrating sphere from the surrounding glass was calculated. The total drag *F*_*2*_ applied to a sphere in a liquid can be calculated as:


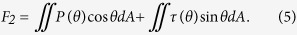


Here, *P*(*θ*) is the pressure applied to the sphere surface obtained by thermal flow analysis and does not include the pressure originating from the interfacial tension, and *τ*(*θ*) is the shearing force. The first and second terms on the right side of the equation are the surface integrals of the pressure drag and of the shearing force, respectively. When metal is solid or is accelerating or decelerating, the shearing force cannot be ignored. However, in this case, the metal in the sphere is liquid and migrates at constant velocity. At the interface between them, the melted metal and glass both flowed at the same velocity; hence, the shearing force is ignored. Total drag is expressed as:


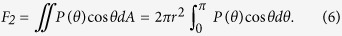


When the migration velocity is constant, the driving force and the pressure drag are balanced, and the migration velocity should be controlled to balance both the forces in the calculation. However, the migration velocity could not be changed during the calculation in the software and the flow velocity was initially set based on the experimental results.

### Driving force

The interfacial tension of the glass decreases with increasing temperature; therefore, the temperature gradient at the stainless steel sphere causes a driving force toward the light source. The pressure caused by the interfacial tension *P*_*i*_(*T*) [Pa] applied to the sphere surface was calculated by the Laplace equation as:


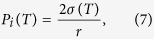


where, *σ*(*T*) [N/m] is the interfacial tension of the glass and stainless steel sphere at temperature *T*.

The driving force applied to the light source *F*_*1*_ is calculated by integrating over the whole sphere surface as:


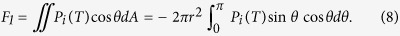


The interfacial tension between the two liquids was estimated using the Girifalco–Good equation^14^. The equation for interfacial tension between the melted glass and stainless steel sphere *σ*_*glass-SUS*_(*T*) was previously derived^15^ (from the Girifalco–Good equation) using the surface tension of glass σ_*glass*_(*T*) and surface tension of stainless steel *σ*_*SUS*_(*T*) as:


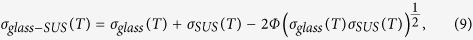


where *Φ* is a parameter relating to wettability. To the best of the authors’ knowledge, the values of *Φ* between borosilicate glass and stainless steel have not previously been reported. In addition, the value of *Φ* is strongly influenced by impurities. For example, the interfacial tension between melted iron and slag (CaO-SiO_2_-Al_2_O_3_) was influenced by the amount of FeO in the slag^16^. In the present study, a stainless steel sphere was melted in silica glass and precipitated as particles; hence, the silica glass around the sphere contained components of stainless steel. Therefore, it was difficult to estimate the value of *Φ*. The interfacial tension *σ*_*glass-SUS*_(*T*) was estimated to balance the driving force *F*_*1*_ with the drag force *F*_*2*_ under any laser power density, with the assumption that the interfacial tension *σ*_*glass-SUS*_(*T*) varied linearly with the temperature. Then, the values were compared with the previously reported results.

### Material parameters

The material parameters are described in the Supplementary information. Specific heat *c*(*T*), thermal conductivity *λ*(*T*), and viscosity *μ*(*T*) were treated as functions of the temperature and approximated from experimental values.

## Results and Discussion

### Migration velocity of the metal sphere

**Figure 2:**
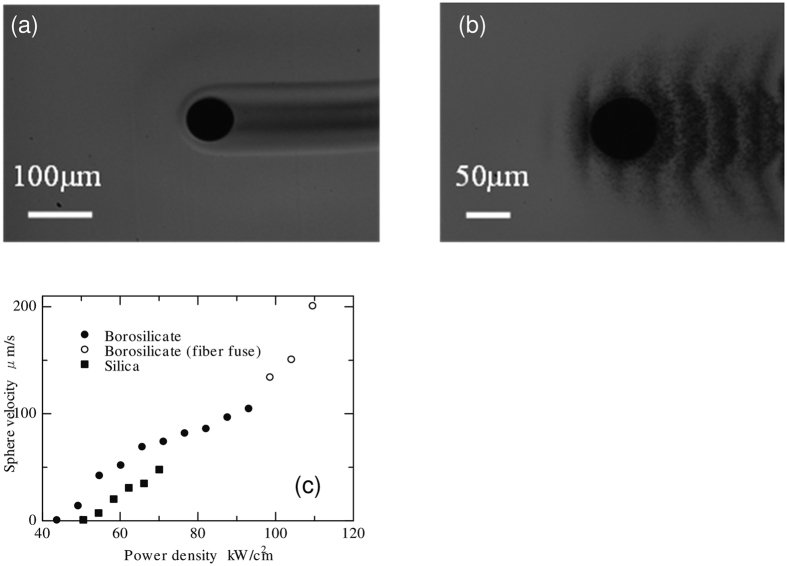
Micrographs of the stainless sphere during migration (**a**) in borosilicate glass and (**b**) in silica glass. (**c**) Influence of laser power density on the migration velocity of the stainless steel sphere.

Laser illumination, with a power of 10 W, induced movement of a stainless steel sphere with a diameter of 80 μm. To simulate the stainless sphere, stainless steel foil was placed on the opposite surface of the glass to the laser illumination. The laser spot diameter at the surface of the stainless steel foil was 150 μm. To measure the migration velocity at a different laser power densities, the laser illumination was paused for each measurement. When the laser illumination was resumed, the sphere began to migrate and achieved a constant velocity within several seconds in the same manner as described in ref. 10. The constant velocity during the sphere migration was calculated from the migrated distance and time obtained from video footage of the migration. Figure 2 shows the sphere in borosilicate glass and in silica glass. In silica glass, black bands were observed around the sphere. These bands consisted of precipitated stainless steel particles with diameters of a few hundreds of nanometres, which was similar to the observations for nickel sphere migration^12^. The precipitation decreased the diameter of the stainless sphere from ~80 μm to ~70 μm for a migration of 2 mm.

The migration velocity was increased by increasing the laser power density, as shown in Fig. 2(c). Migration was observed when the power density was above 49 kW/cm^2^ in borosilicate glass and 55 kW/cm^2^ in silica glass. The maximum velocity for stable migration was 104 μm/s in borosilicate glass and 47 μm/s in silica glass. When the laser power density was increased further, emission was observed from the borosilicate glass. Additionally, the migration velocity increased substantially in borosilicate glass compared with that in silica glass. The emission was caused by the fibre fuse phenomenon, explained as follows. The absorption coefficient of glass increased when the temperature was increased to ~2000 K. A heat spot was formed in the glass, the glass absorbed the laser energy and the heat spot moved towards the light source^17^. The same phenomenon was observed in the bulk glass when illuminated at a high power density^18^,^19^. When this phenomenon is observed, the glass itself absorbs the laser energy. This condition is different from the calculation model where the laser energy is absorbed only at the surface of the sphere. The conditions where the fibre fuse was observed were excluded from analysis in this paper.

### Temperature calculation

**Figure 3:**
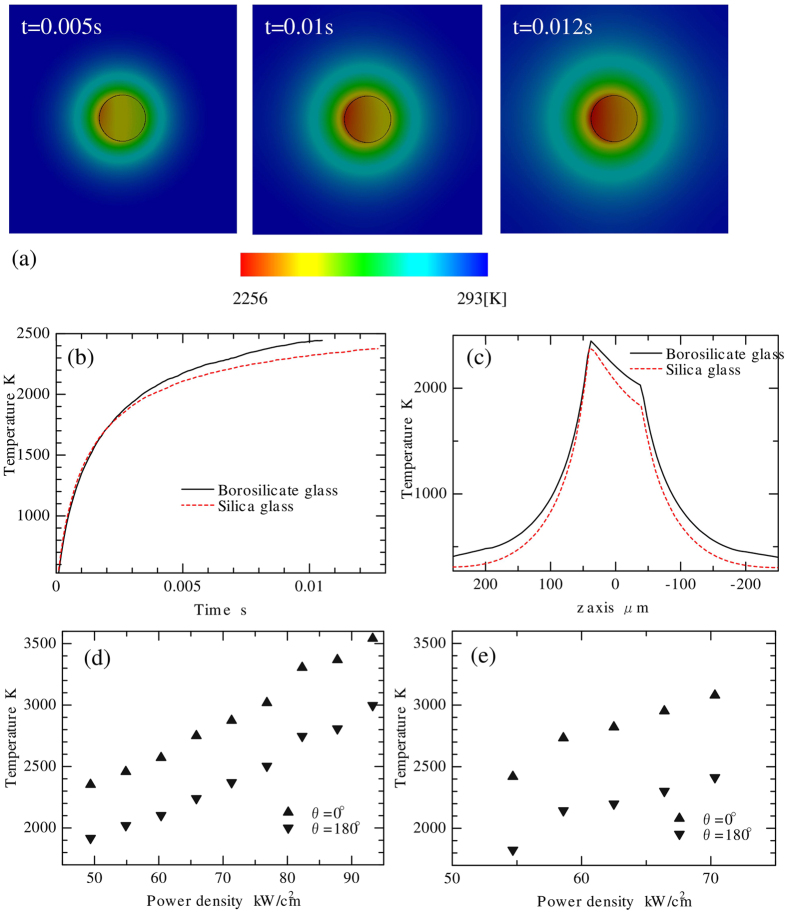
Temperature calculation results of the stainless steel sphere during migration. (**a**) Contour of the temperature of stainless steel sphere in borosilicate glass. (**b**) Temperature change after laser illumination at the surface of the sphere (*r* = 40 μm, *θ* = 0). (**c**) Temperature distribution along the optical axis. (**a**,**b**,**c**) the laser power density was set at 54.9 kW/cm^2^ in borosilicate glass and 54.7 kW/cm^2^ in silica glass. Maximum and minimum temperatures at the surface of the stainless steel sphere with different laser power densities in borosilicate glass (**d**) and in silica glass (**e**).

Typical results are discussed from numerical calculations with a laser power density of 54.9 kW/cm^2^ for borosilicate glass and of 54.7 kW/cm^2^ for silica glass. It was difficult to perfectly match the laser power density applied in each glass in the experiments. Figure 3(a) shows the contour of temperature in the plane containing the optical axis at different time steps. The laser illuminated the samples from the left. Figure 3(b) shows the time lapsed temperature at the region of highest temperature (*r* = 40, *θ* = 0°) until the steady-state temperature (temperature change was less than 1 K for a subsequent time step) was reached. The temperature in both glasses increased upon laser illumination to over 2000 K in 0.005 s and was saturated after 0.01 s at ~2400 K. The temperature distribution along the optical axis (z axis in Fig. 2) is shown in Fig. 3(c). The highest and lowest temperature points at the sphere surface were the laser-illuminated side (*θ* = 0°) and non-illuminated side (*θ* = 180°), respectively. The temperature difference at the sphere surface was ~440 K in borosilicate glass and ~590 K in silica glass.

The temperature distribution was calculated using the same conditions as the experimental conditions. Figure 3(d) and (e) show the highest (at *θ* = 0°) and lowest (at *θ* = 180°) temperatures at the stainless steel sphere surface with different illumination conditions. The temperature difference at the sphere surface was 440–560 K in borosilicate glass and 590–670 K in silica glass.

The temperature at which glass absorption commenced was considered. As shown in Fig. 3(d), the maximum allowable temperature for the sphere migration in borosilicate glass was calculated to be 3540 K when the laser power density was set at 93 kW/cm^2^. When the laser power density was further increased, laser absorption by the glass, which was the same phenomenon as the fibre fuse, was observed. These results indicate that the glass absorption occurred when the temperature was higher than 3540 K. The temperature to ignite the fibre fuse has been estimated to be 3200 K–3700 K previously^20^, which agrees well with our observations.

### Driving force and drag force

**Figure 4:**
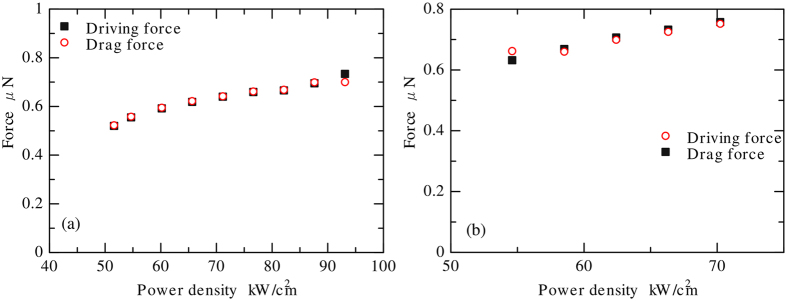
Driving force and drag force applied to the stainless steel sphere with different laser power densities in borosilicate glass (**a**) and in silica glass (**b**).

Figure 4 shows the calculated results of the driving force and drag force. The interfacial tension was supposed to be a linear function of the temperature, and was approximated to balance the driving force *F*_*1*_and the drag force *F*_*2*_ under any temperature. As a result, the interfacial tension was estimated as:


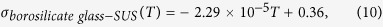



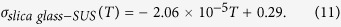


The driving force and drag force balanced well under any laser power density, as shown in Fig. 4; therefore, the supposition that interfacial tension is a linear function of temperature explains the phenomenon well.

The driving force was generated by the non-uniformity of the interfacial tension caused by the temperature difference. The driving force was dominated by the temperature coefficient 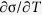 [N/m/K] and the constant term did not affect the driving force; hence, only the temperature coefficient 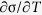 was considered. The temperature coefficients for the glasses were taken as:


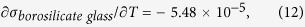



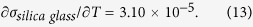


These values were approximated from ref. 21 (glass No. 5, Fig. 7) for borosilicate glass and from ref. 22 for silica glass.

The temperature coefficient of the surface tension of SUS304 was reported as −3.8  × 10^−4^ N/m/K^23^. Butler *et al*. proposed that the surface tension of solutions can be estimated from the function of each consistency^24^. The temperature coefficients of the major components of stainless steel—iron, nickel, and chrome—have been reported as −3.9 × 10^−4^, −3.5 × 10^−4^, and −2.0 × 10^−4^ [N/m/K], respectively^25^. These values were an order of magnitude larger than those of the glasses.

As shown by equation (9), the interfacial tension between glass and stainless steel was calculated by adding the surface tension of glass and stainless steel and subtracting the compensation term. The surface tension of silica glass increased with increasing temperature because the temperature coefficient of silica glass was positive. The temperature coefficient of stainless steel was negative and an order of magnitude larger than that of silica glass; therefore, the surface tension of stainless steel dominated the interfacial tension. The interfacial tension decreased with increasing temperature and caused the driving force to move the stainless steel sphere towards the light source. Few studies have previously reported the temperature dependence of the interfacial tension between metal and glass. Detailed measurement of the interfacial tension is indispensable for the quantitative discussion.

In previous research^10^,^11^, material parameters were treated as constant values, and the tendency of the calculated sphere migration velocity on changing the laser power density did not correlate well with the experimental result. The calculated velocity was 3–160% higher than the experimental result. In this study, the calculated result confirmed the experimental result under any laser power density. Hence, calculation with consideration of the temperature dependence of the material parameters enabled precise calculation. To obtain more accurate results, the interfacial tension estimation and radiation from the sphere needs to be considered.

### Visualisation of glass flow

**Figure 5:**
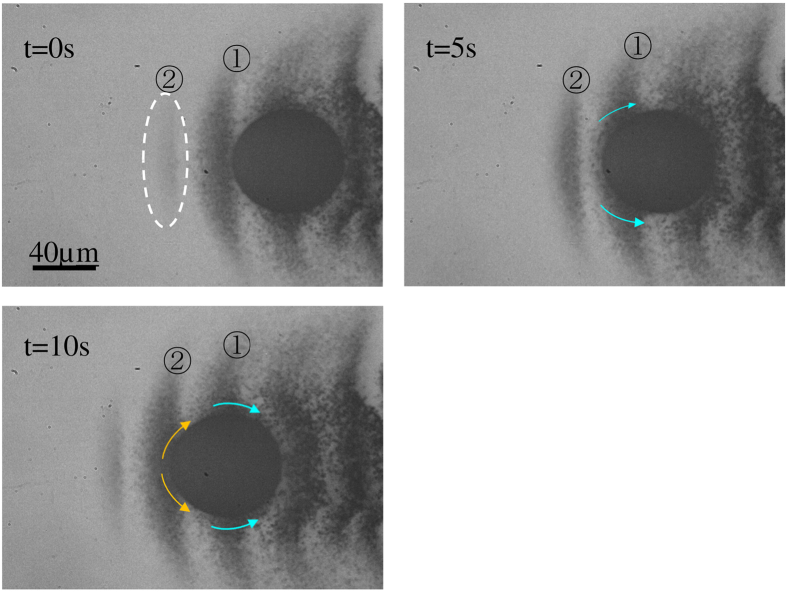
Time lapse images of the precipitated stainless steel particle migration caused by the sphere migration. The laser power density was set at 54.7 kW/cm^2^.

The migration of the stainless steel sphere in silica glass precipitated stainless steel particles around the sphere. The precipitated particles are shown in Fig. 5; the particles were precipitated up to ~60 μm from the sphere in the direction of the sphere migration. The precipitated particles did not migrate by laser illumination. However, the glass flow caused the particles to migrate when the sphere passed nearby.

**Figure 6:**
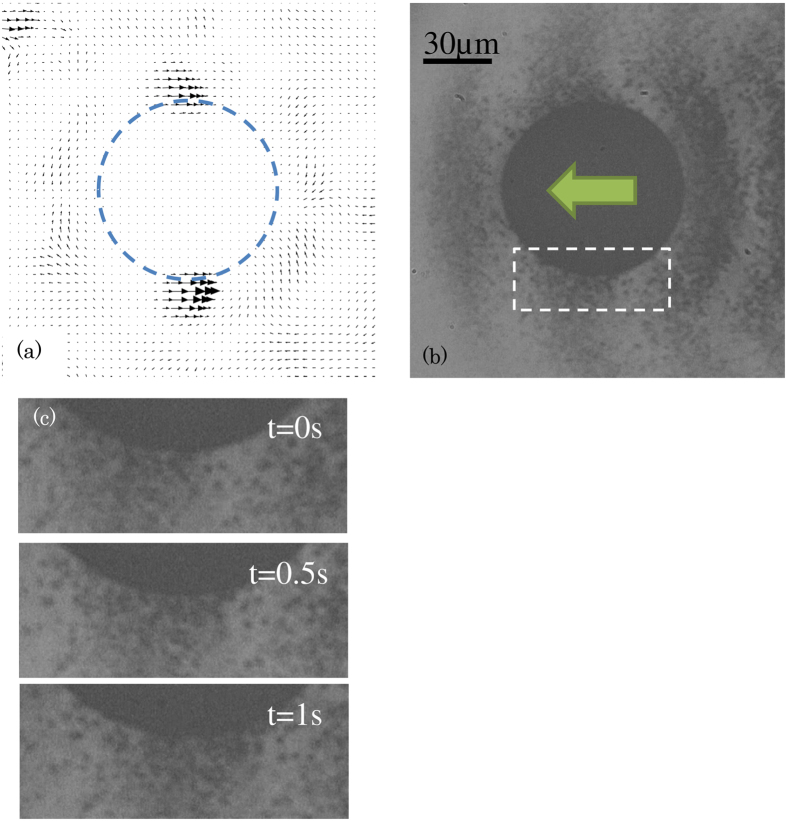
Velocity field of (**a**) glass melt and (**b**) original image used for the velocity field measurement. (**c**) Time lapse magnified image in the area marked by the rectangle in (**b**). The laser power density was set at 58.5 kW/cm^2^.

Figure 6 shows the PIV results of the sphere migration in silica glass. Figure 6(a) is the velocity field; (b) is the photo used for PIV. Figure 6(c) shows magnified time lapse images of Fig. 6(b). The system in Fig. 6(a) was expressed where the sphere was fixed, which was converted from the system used for the experiment where the camera was fixed. Figure 6(c) shows the magnified image of the area marked with a rectangular in Fig. 6(b) where the glass flow was fast. Some precipitated particles were unclear because they were not located on the focal plane. These unfocused particles did not affect the velocity measurement results because the PIV determined the average velocity only in the inspection area.

The PIV did not directly detect the velocity of the glass melt but rather the particle velocity. Therefore, the particle tracking in the glass melt is discussed. The factors affecting the particle tracking in the glass melt are gravity and the rapid velocity change. The effect of gravity has been discussed. The size of the precipitated particles was a few hundred nanometers^12^. The terminal sink velocity *U* by gravity is calculated by the equation:


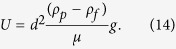


Here, *d* is the diameter of the precipitated particles, *ρ*_*p*_ and *ρ*_*f*_ are the density of the precipitated particles and softened glass, respectively, *μ* is the viscosity of the glass, and *g* is the gravitational acceleration. The calculated terminal sink velocity *U* was in the order of pm/s; hence, the effect of gravity was ignored. The acceleration of the glass flow was calculated to be 2.5 × 10^−4^ μm/s^2^ by assuming that the glass flowed with a velocity of 100 μm/s at the surface of the 80-μm-diameter stainless steel sphere. The acceleration of the glass flow was small; therefore, the precipitated particle tracked the glass melt flow.

### Comparison between experimental results and calculated results

**Figure 7:**
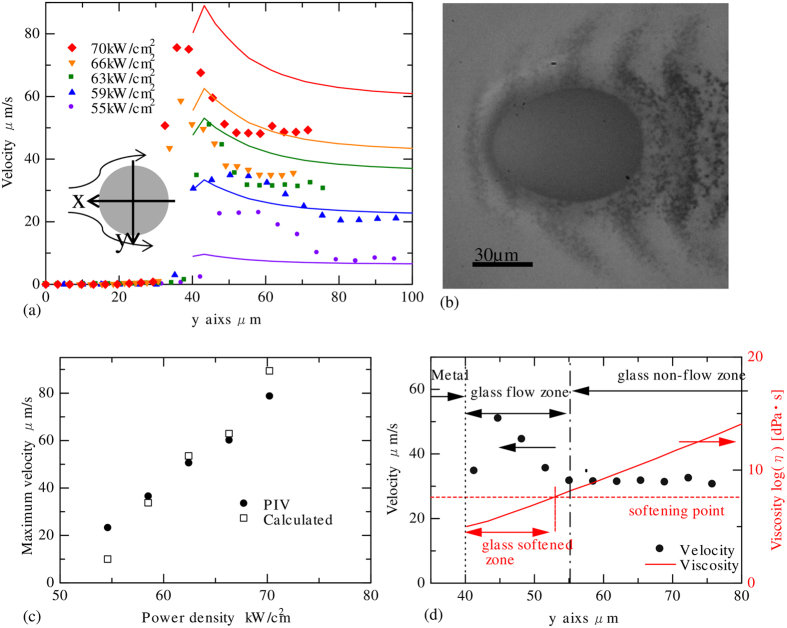
Comparison of the simulated and experimental flow velocity. (**a**) Glass flow velocity along the y-axis with different laser power density. (**b**) Deformed stainless steel sphere migrating at high velocity. The laser power density was set at 70 kW/cm^2^. (**c**) Maximum velocity of the glass melt obtained by PIV and calculated by thermal fluid analysis. (**d**) Comparison between the velocity through the glass melt and viscosity of glass. The laser power density was set at 63 kW/cm^2^.

The velocity of the glass flow along the y-axis (perpendicular to the optical axis) is shown in Fig. 7. The calculated velocity is also shown in Fig. 7. The velocity was expressed as normal in the system where the sphere was fixed; hence, the flow velocity in the area far from the sphere was equal to the velocity of the sphere migration. The maximum velocity around the sphere increased with increasing laser power density.

In the case with a power density of 55 kW/cm^2^, the glass flow velocity was a maximum at y = 47–58 μm, which is 7–18 μm from the sphere surface. The glass flow was not detected at y > 72 μm (more than 32 μm from the sphere surface), which showed that the difference of the velocity from the sphere migration velocity is less than 1.5 μm/s. This result means that the glass flows in the area 32 μm around the sphere. When the laser power density was 63 kW/cm^2^, the glass flow zone was 15 μm from the sphere surface, and the glass flow area shifted to the centre of the sphere in the area of 32–49 μm on the y-axis. The shift was caused by the sphere deforming, elongating into an ovoid, as shown in Fig. 7(b). The reason for the deformation was considered to be that the 80-μm-diameter metal sphere migrates in a narrow softened area of the glass which has a diameter of ~110 μm, at a velocity of 47 μm/s. In addition, inhomogeneity of the interfacial tension and viscosity might also cause the deformation. The maximum velocities obtained from the PIV and from the numerical calculation are shown in Fig. 7(c) and correlate well. This result shows that the thermal flow calculation result is reasonable.

At laser power densities of 55 kW/cm^2^ and 59 kW/cm^2^, the glass flow area was 32 μm around the sphere; this area was larger than that for other laser power densities. With increasing laser power density, the migration velocity of the sphere increased and the thermal diffusion length decreased because the heating time was reduced. In contrast, the temperature of the sphere, which increases with increasing laser power density, may increase the softened area of the glass. Therefore, the glass flow area showed no overall trend with laser power density change.

Glass flows only at high temperatures; hence, the temperature and glass flow area were compared. The glass softening point was defined as the temperature where the viscosity was 10^7.5^ dPa·s. The softening point of silica glass is 1897 K^26^. Figure 7(d) shows the glass flow velocity along the y-axis obtained by PIV; the glass viscosity was calculated from the temperature distribution using Eq. S2 at the laser power density of 63 kW/cm^2^. The migration velocity of the sphere was 32 μm/s, and the glass did not flow in the area of y > 55 μm, where the velocity was constant at 32 μm/s. The glass flowed at y = 40–55 μm. The viscosity of the glass was 10^5^ dPa·s around the sphere (y = 40 μm) and increased with increasing distance from the sphere. The viscosity was 10^7.5^ dPa·s (softening point) at y = 53 μm. The softened area of 13 μm, calculated by thermal flow analysis and the glass flow area of 15 μm, obtained by PIV, matched well. The same calculation was performed under a different power density. The glass flow area was 32 μm, and the softened area was 3 μm at a power density of 55 kW/cm^2^. At the power density of 70 kW/cm^2^, the glass flow was in the area of 16 μm and the softened area was 18 μm. The glass flow area obtained by PIV and the calculated softened area matched well, when the power density was larger than 63 kW/cm^2^. The calculation results properly expressed the temperature distribution. However, both values were substantially different from one another at a power density of 55 kW/cm^2^. The accuracy of the PIV result was confirmed using the original movie of the migration. This mismatch was believed to be caused by factors that had been ignored, such as radiation from the stainless steel sphere and laser absorption by the precipitated particles.

## Conclusion

The velocity of the laser induced stainless steel sphere migration in glass was measured, and the glass flow around the sphere was visualised by PIV. Thermal flow analysis, with consideration of the temperature dependence of material parameters, was conducted. The applied force, induced by the interfacial tension gradient and drag force, was calculated, and the glass flow was simulated. Comparison between the experimental and the theoretical calculation results confirmed that both results were matched well. The following results were clarified.

A SUS 304 sphere with a diameter of 80 μm was implanted into glass. The migration velocity of the sphere increased with increasing laser power density; the maximum velocity was 104 μm/s in borosilicate glass and 47 μm/s in silica glass. The stainless steel sphere was heated up to more than 2000 K; the temperature difference at the surface of the stainless sphere was 450–600 K in borosilicate glass and 580–680 K in silica glass. A temperature gradient of the interfacial tension 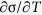 between the stainless steel sphere and the glass was calculated to be −2.29 × 10^−5^ N/m/K in borosilicate glass and −2.06 × 10^−5^ N/m/K in silica glass. The driving force induced by interfacial tension was ~0.7 μN and matched well with the drag force under any laser power density.

The glass flow induced by stainless steel sphere migration was visualised using the precipitated stainless steel particles in silica glass. Glass flowed at a maximum relative velocity of ~80 μm/s with the stainless steel sphere. Glass flowed in the region 15–30 μm from the surface of stainless steel sphere. These results agreed well with the thermal flow analysis.

## Methods

**Figure 8:**
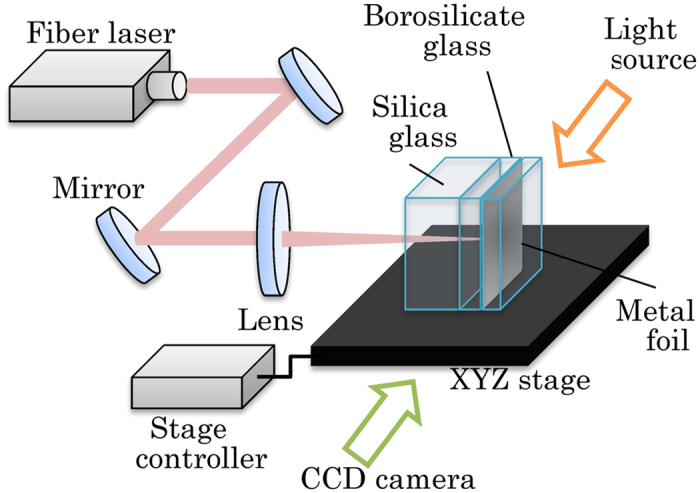
Experimental setup.

The experimental setup is shown in Fig. 8. The configuration was the same as that described previously^12^. Glass plates and stainless steel foil were stacked in the following order: 10-mm-thick silica glass (AQ series, AGC Asahi Glass Co., Ltd., Tokyo, Japan), 5-mm-thick borosilicate glass (Pyrex, Corning 7440, Corning Inc., Corning, NY, USA), 10-μm-thick stainless steel foil (SUS 304, #753173, Nilaco Corp, Tokyo, Japan) and a glass plate to ensure good contact.

A continuous wave fibre laser (RFL-C020/A/2/A, WuhHan Raycus Fiber Laser Technologies Co., Ltd., Hubei, China) with a wavelength of 1064 nm was used. The M^2^ of the laser was <1.1. The laser beam was focused with a lens (NYTL-30-40PY1, Sigma Koki Co., Ltd., Saitama, Japan) and illuminated the stainless foil through the silica glass and borosilicate glass. In this experiment, the laser was diffracted at the surface of the silica glass and the interface between the silica and the borosilicate glasses. By considering the diffraction, a beam spot size (1/e^2^) of ~135 μm in borosilicate glass and ~150 μm in silica glass illuminated the stainless steel sphere.

To visualise the precipitated stainless steel particles, the side-view shadowgraph was obtained with a CCD camera. Metal halide lamp (LS-M250 Sumita Optical Glass Inc., Saitama, Japan) illuminated the opposite side to the CCD camera. A band pass filter (10BPF10-440, Newport Corp., Irvine, CA, USA) was placed in front of the CCD camera to eliminate thermal emission from the stainless steel sphere.

The laser illuminated the stainless steel foil through the silica glass and borosilicate glass. The stainless steel foil was heated and melted and implanted into the borosilicate glass. The stainless steel sphere emitted light and migrated towards the light source, as described previously^9^, and stopped at a certain point because of the defocusing of the laser power density. Then, the illumination was halted and the stage was moved towards the focus. Laser illumination was resumed, and the sphere migrated again. The laser power and sphere position was adjusted to remain within the frame of the side-view CCD camera. By repeating this process, the sphere migrated into the silica glass after passing through the borosilicate glass.

In the silica glass, stainless steel particles with diameters of a few hundred nanometres was precipitated around the sphere at intervals of ~20 μm. To obtain the glass flow velocity field, the migration of the precipitated particles was recorded with the CCD camera. The video of the sphere and precipitated particle migration was processed with JPIV software.

## Additional Information

**How to cite this article**: Hidai, H. *et al*. Experimental and theoretical study on the driving force and glass flow by laser-induced metal sphere migration in glass. *Sci. Rep.* **6**, 38545; doi: 10.1038/srep38545 (2016).

**Publisher's note:** Springer Nature remains neutral with regard to jurisdictional claims in published maps and institutional affiliations.

## Supplementary Material

Supplementary Information
