# Supplementary material for: Experimental and theoretical study on the driving force and glass flow by laser-induced metal sphere migration in glass
Source: Sci Rep. 2016 Dec 9;6:38545. doi: 10.1038/srep38545 (PMC5146938; doi:10.1038/srep38545)
Supplement: Supplementary Information [file srep38545-s1.pdf]

Supplementary Information

Experimental and theoretical study on the driving force and glass flow by laser-induced metal sphere migration in glass

Hirofumi Hidai, Jun Wada, Tatsuki Iwamoto, Souta Matsusaka,

Akira Chiba, Tetsuo Kishi and Noboru Morita

Simulation model

In the analysis, glass was treated as a Newtonian liquid and the viscosity was dependent on the temperature. In the software, multiple fluids could not be handled; hence, the stainless steel sphere was defined as a solid. In the experiment, the stainless steel sphere was confirmed to be melted, but no effect of the flow in the sphere for the calculation of the temperature field. The Péclet number was calculated to be less than 1.5×10^{-3}, and the flow speed was much smaller than heat transfer rate. The Péclet number was calculated by assuming the thermal diffusion coefficient at 2000 K, representative length of 80 μm, which is the sphere diameter, and migration velocity of 100 μm/s.

Figure S1 shows the thermos-fluid analysis model. The centre of the sphere was set as the origin. The diameter of the sphere was set at 80 μm. The computational domain was a cube, and the length of each side was 500 μm, because the thermal diffusion length was calculated to be 162 μm in borosilicate glass and 191 μm in silica glass during a heating time of 1 s. The initial temperature was set at 293 K. The boundary conditions at the surface of the calculation domain were set at insulation requirement. The laser illuminated in the z direction from the left and the heat source was set at the laser illuminated side surface of the sphere, as shown in equations (3) and (4). Glass was treated as a fluid, and the sphere was moved along the z-axis with a constant speed. The migration speed used was obtained from the experimental results. The size of the mesh was set as a cube with the side length of 10 μm. The mesh was separated to 2^4. The time step was set at 1 μs.

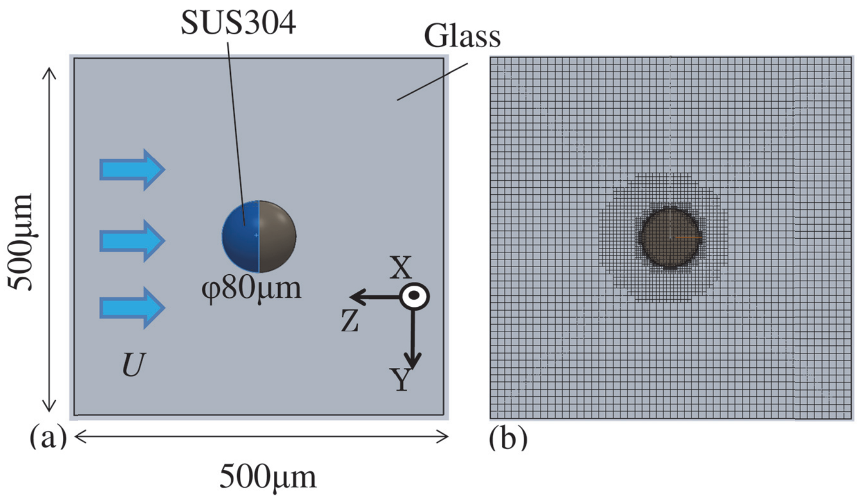

Supplementary Figure S1 Thermal flow analysis model (a) and computational mesh (b).

Material parameters

Viscosity of glasses

The viscosity of glass η(T) [Pa･s] can be approximated with the Vogel–Fulcher–Tammann equation, using the data in Reference [1] as:

$$\log \eta_{borosilicate}(T) = -1.0836 + 5433.1/(T-452)\,, \tag{S1}$$

$$\log \eta_{silica}(T) = -5.6688 + 25172.1/(T-0)\,. \tag{S2}$$

The viscosity was plotted in Supplementary Fig. S2.

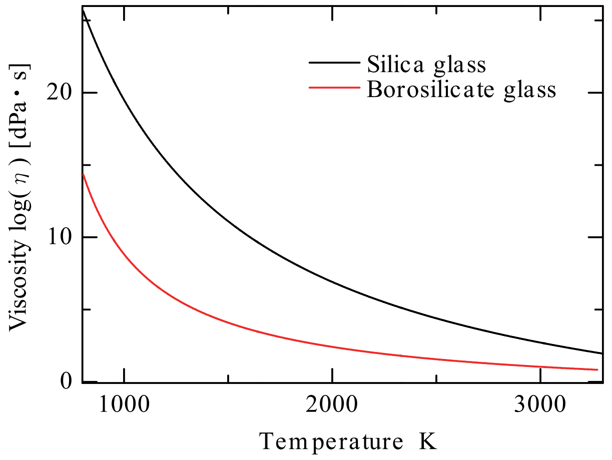

Supplementary Figure S2 Temperature dependence of the viscosity of borosilicate and silica glass.

Coefficient of heat transfer and specific heat of glasses

The coefficient of heat transfer $\lambda(T)$ [W/m/K] and the specific heat $c(T)$ [J/kg/K] of glass were determined using the following equations: for borosilicate glass[2,3],

$$\lambda_{borosilicate}(T) = 0.7711\ln(T) - 3.3545, \quad \text{(S3)}$$

$$c_{boroslilicate}(T) = 403.7\ln(T) - 1534, \quad \text{(S4)}$$

and for silica glass[4],

$$\lambda_{silica}(T) = 1.0866\ln(T) - 5.0964, \quad \text{(S5)}$$

$$c_{silica}(T) = 371.95\ln(T) - 1366.7. \quad \text{(S6)}$$

These equations were approximated from the reported values in the references. The coefficient of heat transfer $\lambda(T)$ and the specific heat $c(T)$, expressed by equations S3–S6, may have negative values, but the equation values were confirmed to have similar values to the reference data over 293 K.

Density

The density of both borosilicate glass and silica glass was set at 2200 kg/m^3.

Material constants of stainless steel

The stainless steel sphere was melted, and the coefficient of thermal conductivity shown in Reference [5] was approximated as:

$$\lambda_{SUS}(T) = 8.35 + 0.0114T. \quad \text{(S7)}$$

The specific heat of the stainless steel was approximated as follows by using the data in Reference [6]:

$$c_{SUS}(T) = 472 + 13.6\times10^{-2}T - 2.82\times10^{6}/T^{2}. \quad \text{(S8)}$$

The density of the stainless steel was set at 7900 kg/m^3.

The absorption coefficient of stainless steel was increased with an increasing temperature. Xie *et al.* reported the reflectivity of 55.83% at the temperature just below the melting point[7]. In this calculation, the reflectivity of stainless steel sphere was set at 60%.

References

1 Pye, L. D., Montenero, A. & Joseph, I. *Properties of glass-forming melts*. (Taylor & Francis, 2005).

2 Pillai, C. G. S. & George, A. M. An improved comparative thermal conductivity apparatus for measurements at high temperatures. *Int. J. Thermophysics* **12**, 563-576 (1991)

3 Campbell, R. C., Beta, I. A. & Manuelian, G. Thermophysical Properties of Pyrex 7740 Glass Over the Temperature Range of -180 ˚C to 475 ˚C. *Proc. 31st Inter. Thermal Conductivity Conf. / Proc. 19th Inter. Thermal Expansion Symp.*, 142 (2013).

4 Kuzuu, N. et al. *Practical manual for amorphous siliceous materials (in Japanese)*. (Realize SE, 1999).

5 Mills, K. C. *Recommended Values of Thermophysical Properties for Selected Commercial Alloys*. (Asm Intl, 2001).

6 Mills, K. C., Su, Y., Li, Z. & Brooks, R. F. Equations for the Calculation of the Thermo-physical Properties of Stainless Steel. *ISIJ Int.* **44**, 1661-1668 (2004).

7 Xie, J. Laser welding of thin sheet steel with surface oxidation. *Welding J.* **78**, 343-348 (1999).
